# Effectiveness of Dialysis in Psoriasis: A Short Review

**DOI:** 10.7759/cureus.30061

**Published:** 2022-10-08

**Authors:** Pooja Pandey, Sunil Kumar

**Affiliations:** 1 Department of Medicine, Jawaharlal Nehru Medical College, Datta Meghe Institute of Medical Sciences, Wardha, IND

**Keywords:** kidney function, end stage kidney disease (eskd), chronic kidney disease (ckd), non-uremic patients, renal failure, impacts, peritoneal dialysis, haemodialysis, dialysis, psoriasis

## Abstract

Psoriasis is a chronic, incurable condition with an erratic course of symptoms and triggers by nature. Psoriasis patients need medical attention that extends beyond only treating skin conditions and joint issues. Because psoriasis is so complex, treating it with medication alone does not work well; comprehensive, whole-person treatment is required. Screening for concomitant diseases including hypertension, dyslipidemia, diabetes mellitus, cardiovascular issues, and their adverse effects like myocardial infarction and stroke is a part of treating psoriasis. Regular screening for these linked illnesses should be done. Essential elements of psoriasis care include co-medication to avoid drug interactions or drug-induced psoriasis, as well as the identification and management of trigger factors. The lack of widely used and established diagnostic criteria restricts these studies. Essential elements of psoriasis management include routine screening for these associated disorders, co-medication to avoid drug interactions or psoriasis caused by drugs, as well as the identification of trigger factors and their management. This short review highlights the effectiveness of dialysis in people with psoriasis and the fact that the benefit is more pronounced with peritoneal dialysis than with hemodialysis.

## Introduction and background

Psoriasis is a chronic, non-communicable illness that affects sufferers' quality of life (QoL) significantly and is unpleasant, disfiguring, and crippling. Although it can happen at any age, the 50 to 69 age range has the highest prevalence [1-3]. Psoriasis is a severe global issue as stated country prevalence rates range from 0.09% to 11.4% [4-5]. Descriptive cross-sectional studies demonstrated that the probability of developing chronic kidney disease (CKD) increased with the severity and duration of psoriasis in psoriatic patients. In this investigation, 10 CKD patients had psoriasis that had been present for more than 10 years. Psoriasis's cause is still unknown, despite signs of genetic susceptibility. According to a study at a university hospital, 136,529 individuals with mild psoriasis and 7,354 patients with severe psoriasis were matched to 689,702 unaffected volunteers based on their treatment histories [6]. Additionally, both internal and environmental causes, such as minor trauma, sunburn, stress, systemic medications, and infections can all contribute to psoriasis [7-10]. Psoriasis is a skin and nail condition that coexists with a number of other diseases. Skin lesions that are localised or widespread are red papules and plaques that are typically symmetrical, clearly defined, and covered in either white or silver scales [11-13]. Pain, stinging, itching, and psoriatic arthritis (1.3%-34.7%) are usual in those with psoriasis. The cross-sectional study found that the total prevalence of CKD was 15.2%. (1.4% for those aged 20 to 39; 9.4% for those aged 40 to 64; and 38.1% for those over 64) [14]. Nail alterations occur among 4.2% and 69% of all psoriasis patients. Psoriasis sufferers are said to be more susceptible to acquiring other serious clinical conditions like cardiovascular disease and other noncommunicable diseases (NCDs) [15-18]. Psoriasis is extremely taxing on the body, the mind, and society. Patients who only had skin problems had a CKD prevalence of 9.5%, whereas those who also had psoriatic arthropathy had an 18.8% prevalence. In general, QoL is frequently seriously compromised. For those who have psoriasis, deformity, incapacity, and a significant reduction in productivity are common problems [19-22]. Additionally, there are major costs associated with maintaining mental health, such as greater rates of depression that have an adverse effect on both individuals and society [23-24]. For people with psoriasis and their families, social exclusion, prejudice, and stigma are mentally damaging. The exclusion is largely due to society's response to psoriasis, and this can change [25-31].

Psoriasis prevalence estimates on a global scale are being provided by an increasing number of population-based research [32-35]. The prevalence of psoriasis varies around the globe. Prevalence in various communities ranges from 0% to 11.8 %, according to published reports [36-38]. The range for the majority of the data is from roughly 0.5% to almost 2.5%. Psoriasis prevalence was estimated to be 4.7% in Canada and 4.6% in the USA, respectively. Europe's data show little difference among nations, with rates like 1.45% (Croatia), 1.60% (Norway), and 1.4% (UK). Only 0.7% of people in East Africa and 0.7% of people in China's Henan district were determined to be affected [39-41].

According to their treatment preferences, 136,529 persons with mild psoriasis and 7,354 people with severe psoriasis were matched with 689,702 unaffected people in a population-based cohort analysis and nested cross-sectional study carried out in the UK. In spite of the absence of the typical risk variables, these analyses found that moderate to severe psoriasis increased the likelihood of chronic renal failure. Regardless of known risk variables, they arrived at the conclusion that moderate to severe psoriasis is linked to moderate to advanced chronic renal disease [42].

Additionally, it has been established that adults with psoriasis are more likely to experience end-stage renal failure and chronic kidney disease. Psoriasis and renal disorders have frequently been documented to coexist in recent years, resulting in the term “psoriatic nephropathy’’ [43]. Men and women started at almost the same time on average, and the most common age range was 20 to 39 years. In a different study from North India, 0.8% of skin patients had psoriasis, but the sample size was quite small [44-45]. There was a 2.5:1 sex ratio (men to women). This study found that the mean onset ages for women were lower than those for men. The prevalence of psoriasis among dermatology outpatients was estimated to be 2.8% (530 persons) in a later study with larger sample size, while the male-to-female ratio remained unaltered [45].

This review article highlights the effects of dialysis in patients with kidney disease and with normal kidney functions having psoriasis.

## Review

Methodology

**Figure 1:**
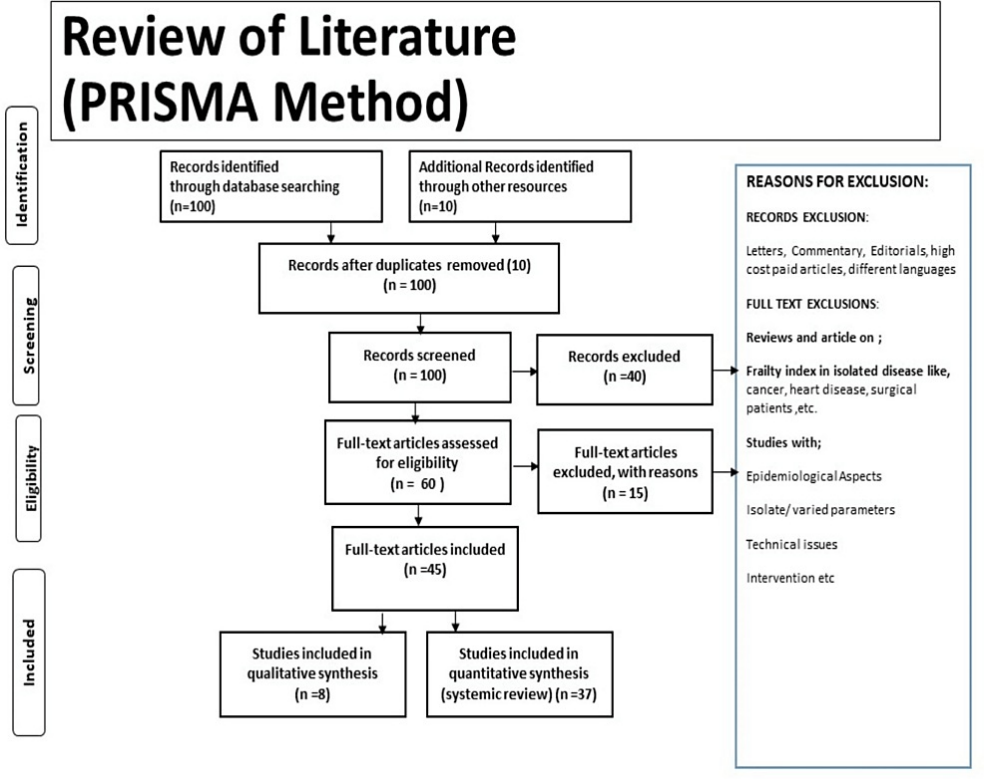
Research methodology by PRISMA methods. Dialysis in psoriasis and its role to halt the progression of psoriasis with and without kidney disease; studies from the last ten years; studies devoted entirely to the treatment of psoriasis with haemodialysis in patients with and without kidney diseases

The following is being explored in this review: dialysis in psoriasis and its role to halt the progression of psoriasis with and without kidney disease. A literature search in English was conducted using the electronic databases PubMed, Medline, Embase, and Google Scholar. The search terms were psoriasis and CKD or Renal Disease or Dialysis or Haemodialysis or Peritoneal Dialysis. The archiving of relevant papers was supported by the writers' personal knowledge and experience in the field. Articles that matched the following criteria were included in this review: studies in English; studies from previous years; and studies devoted entirely to the treatment of psoriasis with haemodialysis in patients with and without kidney diseases. The research methodology by the Preferred Reporting Items for Systematic Reviews and Meta-Analyses (PRISMA) method is shown in Figure 1.

Pathophysiology

**Figure 2:**
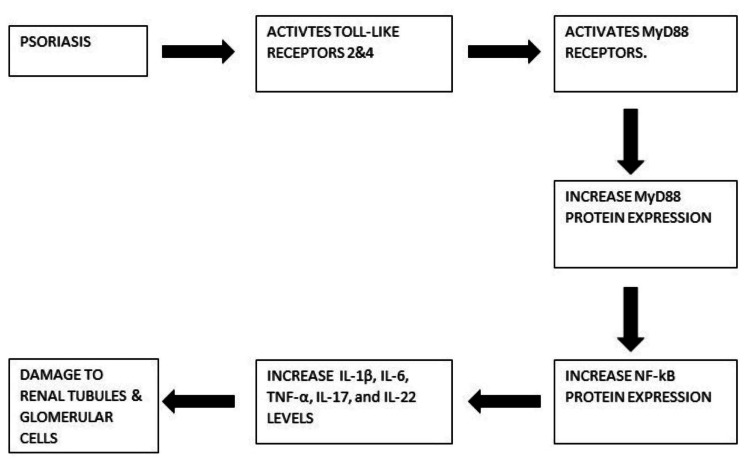
Illustrating the suggested process of kidney injury in psoriasis. Psoriasis activates Toll-like receptors 2 and 4, which further activates MyD88 receptors, which increases MyD88 protein expression, which increases NF-kB protein expression, ultimately increasing IL-1β, IL-6, TNF-α, IL-17, and IL-22 levels, which damages renal tubules and glomerular cells [3-4,27,30].

According to a population-based cohort study, moderate to severe psoriasis increases the risk of chronic renal illness without being connected to any known risk factors [4]. T lymphocyte dysfunction and glomerular damage are both caused by immune system malfunction in psoriatic patients. Glomerular damage can be brought on by autoimmune diseases. Last but not least, several medications used to treat psoriasis can be harmful to the kidneys [27]. Furthermore, non-steroidal anti-inflammatory drug users are more likely to experience renal issues. It is yet unclear what pathophysiological factors have a relation between people with severe psoriasis and their susceptibility to acquiring end-stage renal failure and chronic kidney disease. The fact is that major arteries' related inflammation has been recognised as a hallmark of the chronic inflammatory illness in psoriasis [3]. Psoriasis is a skin condition that causes inflammation, but recent research has shown that it is also associated with kidney illness because it increases the level of albumin excretion in the urine, which increases the chance of developing long-term kidney disease. A study [30] found that patients with severe psoriasis had a tripled risk of end-stage renal disease and a doubled risk of incident chronic kidney disease when compared to controls. The suggested process of kidney injury in a psoriasis patient is shown in Figure 2.

Discussion

Men are twice as likely to develop psoriasis as women, and the prevalence of the condition ranges from 0.44% to 2.8% in India. The lack of widely used and established diagnostic criteria restricts these studies. Furthermore, there is virtually no accurate data on the disease's historical trends. There is a link between renal illness and psoriasis as elaborated in Table 1.

**Table 1:** The most significant research demonstrating the link between renal illness and psoriasis. ESRD = end-stage renal disease; CKD = chronic kidney disease; GFR = glomerular filtration rate.

Author	Research plan	Result
Wan et al. (2013) [4].	Cohort research	Independent of conventional risk variables, moderate to severe psoriasis is linked to a higher risk of CKD. Age raises the overall likelihood of developing psoriasis-related chronic renal disease, which has therapeutic implications.
Chi et al. (2015) [30].	Cohort research	People with severe psoriasis had twice the risk of incident CKD and three times the risk of incident ESRD compared to those without the condition.
Gonzalez-Parra et al. (2016) [15].	Systematic review	Every year, psoriatic patients should have their creatinine, GFR, and urine albumin tested, especially if they take nephrotoxic medications.
Visconti et al. (2016) [12].	Review article	The extent of the skin lesions determines the classification of psoriasis. Additionally, psoriasis medication might harm the kidneys.
Al-Harbi et al. (2017) [12].	Experimental study	A major factor in the renal dysfuction brought on by psoriasis is oxidative inflammation. Nitric oxide synthase is involved in the renal impairment brought on by psoriasis.
Yu et al. (2017) [12].	Retrospective cohort study	Psoriasis is a separate risk factor for ESRD and chronic renal failure.
Lee et al. (2019) [21].	Cohort research	ESRD was more likely to occur in people Those who had systemic psoriasis were not affected by psoriasis or psoriatic arthritis medication.
Ren et al. (2020) [12].	Review article	Inflammatory cytokines were expressed as a result of psoriasis, harming podocytes.

A study at Penn State University titled 'Moderate to severe psoriasis linked to chronic kidney disease" [26] found that people who are affected by psoriasis on more than 3% of their bodies are more at risk for developing kidney diseases. More importantly, regardless of other risk factors that contributed to the condition like diabetes, hypertension, or usage of NSAIDS, moderate to severe psoriasis raises the likelihood of developing CKD or end-stage renal disease (ESRD). The article then suggested that physicians (not just dermatologists or epidemiologists) needed to screen their patients for kidney disease by administering simple blood and urine tests for kidney disease and to make sure not to prescribe medications that could hurt the patient's kidneys. 

The research team compared 689,702 unaffected people to 7,354 patients with severe psoriasis and 136,529 patients were recorded with intermediate psoriasis. Additionally, they looked at the prevalence of chronic renal illness in 9,000 psoriasis patients who were being monitored prospectively through the electronic medical record. Following a seven-year period of observation, the chance of developing chronic renal illness was greater in people with psoriasis than in the control group. Patients with severe psoriasis were twice as likely to develop CKD and four times more likely to experience renal failure and a need for dialysis. One can still get CKD if only 3% of their skin is damaged by psoriasis. A person is even more likely to develop kidney disease if 10% of their skin is afflicted. Many people with moderate to severe psoriasis undergo treatment with drugs like methotrexate or cyclosporine. However, these drugs can have kidney-related adverse effects [19]. Further research is required to assess which drugs are most effective at reducing inflammation and delaying or preventing renal damage.

Reduced immunoglobulin G (IgG) levels, theorised elimination of chemicals that promote development from the bloodstream, factors connected to psoriasis, polymorphonuclear leukocyte activation, obstruction of neutrophil migration, and increased levels of fibronectin are some of the factors which may have connections with CKD.

On the other hand, several pieces of research link the onset of psoriasis to dialysis-induced growth factors, cytokines, and chemokines. Chronic renal disease is independently predicted by severe psoriasis. Studies that concentrate on elements that both illnesses have in common may be instructive [19-22].

Dialysis’s Effects on Patients With and Without Kidney Disease Who Have Psoriasis

Three psoriasis patients who had failed every form of conventional therapy endured 32 hours of peritoneal dialysis per week for 10 weeks. Eighty percent of the psoriatic lesions in two patients disappeared following the course of treatment. The lesions returned in one of these patients two months later, whereas the other patient is still in relative remission a year later. In the third patient, 50% of the lesions cleared up, but two months after the end of the treatment, the lesions returned. This experience and a literature review suggest that dialysis may benefit people with psoriasis and that the benefit is more pronounced with peritoneal dialysis than with haemodialysis [23-25].

One hundred and fifty of the 944 dialysis facilities in Europe reported dealing with psoriasis patients. Ninety-three centres responded to specialised questionnaires on 97 patients with end-stage renal failure (ESRF) and 49 dialyzed for psoriasis patients with normal renal function (NRF patients). Both the patients and "objective criteria" subjective perceptions indicated that 17 out of 27 NRF patients had improvements in their skin conditions. However, the majority of these patients had just been on dialysis for 9.9 +/- 11.1 months, which is too little time for psoriasis to spontaneously recur. Those with ESRF, on the other hand, had been receiving dialysis for an average of 45 +/- 3.1 months. After starting dialysis, 20% of these individuals' skin conditions significantly improved. This percentage exceeds the anticipated rate of spontaneous long-term psoriasis remission [25,36].

Continuous ambulatory peritoneal dialysis (CAPD) was used to treat four psoriasis patients. While the other two were only having treatment for their psoriasis and had normal renal function, the other two were receiving treatment for renal failure. Three to four daily exchanges throughout prolonged therapy (more than or equal to 12 weeks) may be necessary to achieve an initial full remission. To prevent relapse, ongoing counselling could also be required. As a result, CAPD offers hope for the research of psoriasis and may serve as a last-ditch remedy for very debilitating cases. Because there is a greater chance of infection, psoriasis therapies have been deemed by experts to be too risky for use in individuals with kidney disease. However, three individuals were given ustekinumab treatment by Japanese experts [41-43]. All patients saw improvement in their psoriasis symptoms, and the majority of them accepted the medication well. The patients had previously undergone failed treatments with methotrexate, retinoids, and cyclosporine. Ustekinumab was administered to the three patients for 12 months while they underwent haemodialysis, and all three experienced "rapid and maintained improvement in psoriasis" as a result. Ustekinumab may be a suitable treatment for psoriasis patients who also need haemodialysis, according to these data. However, one patient stopped receiving treatment as a result of an increase in the inflammatory marker C-reactive protein (CRP), which suggested a potential infection. Throughout their first year of treatment, the two surviving patients did not report any negative side effects [42]. The authors stated that recently, biologic therapies had been acknowledged as having sufficient efficacy for severe psoriasis with low occurrence of organ toxicity. Because of this, biologic therapy might be more effective for haemodialysis patients, although the evidence is insufficient.

Our findings need to be verified, the processes causing renal insufficiency in psoriasis need to be discovered, and further research is needed to determine how treating psoriasis affects the risk of developing chronic kidney disease. Another investigation into how psoriasis and CKD are related is being carried out in a tertiary medical centre in Kerala, India. According to a study, psoriatic people have a higher chance of developing CKD the more severe and persistent their psoriasis is. In this study, there was a positive correlation between the duration of psoriasis and the beginning of CKD [3].

Similar results, i.e., causally attributable renal involvement in patients with psoriasis and factors affecting the same, were seen in an Indian case-control study. After ruling out any secondary causes of renal disease, fifty individuals with verified psoriasis were included, and they came to the following conclusion: The inflammatory milieu was implicated by the positive connection between renal involvement and high-sensitivity CRP (hs-CRP) [21]. According to their need for phototherapy or systemic treatment (six months) and/or psoriatic arthropathy, individuals aged 20 or older who had been diagnosed with moderate to severe psoriasis underwent a cross-sectional study. Between October 2007 to May 2018, participants were gathered from a tertiary university hospital's dermatological and rheumatology outpatient clinic. Patients with psoriasis that ranged in severity from moderate to psoriatic arthropathy had a significant frequency of CKD. Having hypertension and being older than 64 may indicate a higher risk of developing CKD. In other words, psoriasis may make individuals with traditional cardiovascular risk factors more likely to develop renal impairment, and the presence of psoriatic arthropathy may raise the risk even further. In these patients, mildly reduced eGFR (estimated glomerular filtration rate) was very common and could raise cardiovascular risk. Our findings imply that in situations of moderate to severe types of psoriasis, renal and cardiovascular risk factors should be taken into account [21].

Following a report on the accidental disappearance of psoriasis lesions after haemodialysis in 1976, numerous minor investigations followed, each claiming a different outcome. Twardowski additionally treated a non-uremic patient with haemodialysis for psoriasis [40]. IgG deposits considerably diminished in all the groups following therapy, although tissue or plasma zinc or copper levels remained the same. Seven patients with severe psoriasis who participated in a controlled trial of haemodialysis, on the other hand, found no appreciable improvement in terms of objective outcomes. Three subjects underwent hemodiafiltration over the course of a day, then again four weeks later. In a second trial, six out of 10 patients (or 60%) in the peritoneal dialysis group and four out of eight patients (or 50%) in the haemodialysis group had improved after six months. One patient saw a transient benefit and 11 days after starting haemodialysis, exfoliative dermatitis appeared. Peritoneal dialysis was not well tolerated nor beneficial for three of the patients. Although the precise mechanism of action of this technique is unclear, it is hypothesised that it works by removing growth factors from the bloodstream, increasing fibronectin levels, and decreasing IgG levels. Dialysis may be beneficial for some psoriasis patients with renal disease; severe psoriasis also predicts CKD. When receiving chronic haemodialysis for renal disease, it is possible for psoriasis to recur, get worse, or develop from scratch. Both haemodialysis and peritoneal dialysis may result in new-onset psoriasis, and variables such as psoriasis development have been connected to cytokines, chemokines, and dialysis-induced growth factor. Leukapheresis and real and fake plasma pheresis were found to have no beneficial benefits in a controlled study. To put it simply, forced osmotic diuresis is useless [40]. Peritoneal dialysis may improve psoriasis outcomes for all patients, but it is never chosen until it is required for its clearly defined goals. Essential elements of psoriasis care include co-medication to avoid drug interactions or drug-induced psoriasis, as well as the identification and management of trigger factors.

Reason to Change From Conventional Psoriasis Treatment

Psoriasis is a chronic, incurable condition with an erratic course of symptoms and triggers by nature. Since the result is frequently lifelong treatment, all treatments must adhere to strict standards and be both highly effective and long-term safe. Treatment for psoriasis is only available to manage symptoms because the cause is still unknown. Treatment options include a wide range of topical and systemic medications as well as phototherapy. Treatment is also necessary to minimise the pain and disability brought on by arthritis. Psoriasis patients need medical attention that extends beyond only treating skin conditions and joint issues. Because psoriasis is so complex, treating it with medication alone does not work well; comprehensive, whole-person treatment is required. Screening for concomitant diseases including hypertension, dyslipidaemia, diabetes mellitus, cardiovascular issues, and their adverse effects like myocardial infarction and stroke is a part of treating psoriasis. Sadness, anxiety issues, and suicidal thoughts are more prevalent among psoriasis patients.

## Conclusions

In this review article, the relationship between psoriasis and kidney illness was investigated. It has been suggested that psoriasis is a systemic condition rather than only a skin condition. In comparison to controls, patients with psoriasis frequently have hyperlipidaemia, hypertension, coronary artery disease, type 2 diabetes, and increased body mass.

Psoriasis's pathophysiology is not entirely known. Previously, it was thought that it was solely brought on by keratinocyte hyperproliferation, but the positive therapeutic effects of immune system-targeting medications today show that there may be an immunoregulation problem. An elevated risk for chronic renal disease is brought on by moderate-to-severe psoriasis and is a separate risk factor. The persistent inflammatory condition that steadily damages all organs, including the kidneys, appears to be linked to the onset of chronic renal failure in psoriasis. Psoriasis and end-stage renal disease are believed to share a number of pathogenetic pathways. Various cytokines, including IL-17, and reactive oxygen species are among them, and psoriasis medications. All of these pathways appear to lead to kidney damage by injuring podocytes. So far, this review article's explanation of the connection between psoriasis and kidney illness has focused on cytokines and reactive oxygen species. There have to be further investigations before a conclusive meta-analysis can be made about the relationship between psoriasis and kidney damage.
